# The effects of Growth hormone treatment discontinuation in Adults on Metabolic profile, Body composition and quality Of Life (GAMBOL Study)

**DOI:** 10.3310/nihropenres.14030.1

**Published:** 2025-09-19

**Authors:** Sherwin Criseno, Anne Topping, Niki Karavitaki

**Affiliations:** 1 Department of Metabolism and Systems Science, University of Birmingham College of Medical and Dental Sciences, Birmingham, England, UK; 2 Centre for Endocrinology, Diabetes and Metabolism, Birmingham Health Partners, Birmingham, UK; 3 Department of Endocrinology and Metabolism, University Hospitals Birmingham NHS Foundation Trust, Birmingham, England, UK; 4 School of Nursing and Midwifery, University of Birmingham Institute of Clinical Sciences, Birmingham, England, UK

**Keywords:** Growth hormone, adult growth hormone deficiency, growth hormone treatment, growth hormone treatment discontinuation, feasibility study

## Abstract

**Background:**

In adults, treatment of growth hormone deficiency with daily recombinant human growth hormone injections has shown to improve many clinical features associated with GHD. Currently, many adults with GHD receive GH treatment indefinitely. However, to date, no study has consistently demonstrated that GH treatment has a sustained beneficial effect in adults with GHD. A randomised controlled trial is needed to understand the impacts of discontinuation of long-term GH therapy in adults. However, prior to embarking on an RCT, the feasibility of a discontinuation study and the acceptability of randomisation to patients and clinicians need to be assessed.

**Research aims:**

(a) to explore the current practice of offering discontinuation of long-term GH treatment in adults in the UK and

(b) to assess the feasibility of conducting an RCT looking at the effects of discontinuing long-term GH treatment in adults with GHD

**Methods:**

This mixed-method study which will be conducted in three phases.

- Phase 1: An online survey of endocrine clinicians’ practice of offering discontinuation of long-term GH therapy in adult patients with GHD.
- Phase 2: Feasibility cohort study which involves recruiting two groups of adult patients with GHD (aged more than 25 years), who have been on GH treatment for at least 5 years.: (a) an intervention group consisting of 20–25 patients who will be discontinuing their long-term GH treatment for two years and (b) a control group consisting of 20–25 patients who will continue with their GH treatment.
- Phase 3: Qualitative study. 10–16 participants will be recruited to explore their experiences of participation in the feasibility study using semi-structured interviews.

**Potential benefits:**

This study will provide evidence of current GH treatment discontinuation practice in the UK and determine the feasibility of any future RCT. Long-term, this could promote and underpin the development of the much-needed relevant clinical guidance.

## Plain Language Summary

Growth hormone deficiency (GHD) is a condition that develops when the pituitary gland secretes less growth hormone (GH) than the body needs. In adults, GHD usually occurs when the pituitary gland becomes damaged by a tumour (growth), surgery and/or radiotherapy. Studies of short duration have shown that treatment with daily GH injections can improve the symptoms experienced by patients with GHD. In the UK, most adults with GHD are prescribed with GH indefinitely. However, some adult patients have questioned whether discontinuing their injections has any negative effects; currently, this is unclear. Hence, a systematic investigation examining the impact of discontinuing long-term GH treatment is urgently needed.

The aim of this is study to assess the feasibility of conducting a large-scale study to compare the effects of continuing and discontinuing long-term GH treatment in adult patients with GHD. This study will be conducted in three phases:

- Phase 1: Online survey of endocrine specialists based in UK endocrine centres treating adult patients with GHD.
- Phase 2: Feasibility study involving two groups of adult patients with GHD who have been receiving GH treatment for at least 5 years. One group (intervention) will include 20–25 patients who are willing to stop taking GH treatment for two years. The second group (control) will include 20–25 patients who wish to continue GH treatment and are willing to undergo study monitoring for two years.
- Phase 3: Qualitative study involving face-to-face or telephone interviews of 10–16 patients to explore their experiences of participating, completing and/or withdrawing from the study.

A patient and public advisory group has helped design this proposal and will be involved throughout the research project. The results of the study will be submitted for publication targeting leading endocrine journals will also be presented at local, national and international conferences and patient support group meeting.

## Introduction

Growth hormone deficiency (GHD) in adults is caused by decreased secretion of growth hormone (GH) from the pituitary gland ^1, 2^. This condition is usually caused by a tumour, surgery and/or radiotherapy involving the pituitary gland ^3^. Adults with GHD present with a constellation of several non-specific features including low mood, poor general well-being, reduced bone mineral density (BMD), increased body fat, increased cholesterol level and reduced exercise capacity ^3– 5^. Treatment with recombinant human growth hormone (GH) injection has been shown, in short-term studies, to improve the wide spectrum of health issues associated with GHD ^6^.

In the UK, the National Institute for Health and Care Excellence (NICE) recommends treating adult patients with GHD with GH until peak bone and muscle mass have been achieved (estimated to be at around the age of 25 years) ^7^. For adults over 25 years old, the prescribing of GH treatment is based on NICE criteria that requires patients to: a) have severe GHD, defined as peak GH response of less than 9 mU/litre during a GH stimulation test; b) have a perceived impairment of quality of life (QoL), as demonstrated by a score of at least 11 in the ‘Quality of Life - Adult Growth Hormone Deficiency Assessment’ (QoL-AGHDA) questionnaire; c) be receiving appropriate treatment for any other pituitary hormone deficiencies. The UK population of adult patients with GHD receiving GH is quite unique and homogenous as they all have severe GHD and poor quality of life (QoL) prior to commencing the treatment ^4^. The UK is the only country in the world that offers GH to adult patients with GHD only after poor QoL scores have been confirmed by using the QoL-AGHDA questionnaire ^1, 4, 6^.

To date, the optimal duration of GH replacement therapy in adults and the consequences of treatment withdrawal have not been established. There are only a few, mainly short-term, follow-up studies that provide conflicting results. Overall, the beneficial effects of GH treatment appear to occur during the first year of therapy with only a few studies reporting sustained benefits up to 5 years ^5, 8– 10^. It is well established that GH secretion decreases in a normal population with advancing age and that low GH levels are even associated with increased longevity and decreased morbidity ^11, 12^. Currently, many adults with GHD are receiving GH indefinitely without knowing if continuing or discontinuing long-term treatment has sustained beneficial or even adverse effects ^13, 14^. Similarly, reliable data on the impact of discontinuing long-term GH therapy in adults is not available.

In the UK, provided that a 7-point improvement (or higher) on the 25-point QoL-AGHDA scale is demonstrated during the first 9 months of GH therapy, adult patients with GHD can continue with their GH treatment long-term ^7^. However, it remains uncertain if the beneficial effects of GH therapy are sustained throughout adult life, as robust evidence on the risks and benefits of long-term GH treatment is limited. Furthermore, the evidence of improved QoL in adult patients with GHD having GH treatment over placebo has not been established ^6^. Anecdotally, clinicians report encountering patients who question the merit of continuing with long-term GH treatment. Paradoxically, the same patients are apparently reluctant to stop GH therapy due to fears of return of symptoms and uncertainty about the short-term and long-term effects of treatment discontinuation. Consequently, and in the absence of clear evidence and definitive guidance, replacement therapy with GH continues indefinitely in many adults.

## Rationale

Many questions on the effectiveness of long-term treatment with GH in adults remain unanswered including its optimal duration and the consequences of treatment discontinuation. The primary aim of this study is to assess the feasibility of conducting a large-scale multi-centre study to evaluate the effects of discontinuation of long-term GH therapy on the metabolic profile, body composition and QoL in a cohort of adult patients with GHD (aged 25 years and over) over a period of 24 months.

Currently, much of the evidence on safety-related issues associated with long-term GH therapy comes from uncontrolled retrospective, and observational studies, which are methodologically inferior compared with randomised control trial (RCT) ^1, 6^. A critical assessment of data from limited RCTs did not show consistent beneficial effects of GH therapy in adult patients with GHD compared with placebo ^6^. In 2016, several international endocrine societies collectively highlighted the need for carefully designed and rigorously conducted cohort studies to monitor the safety of long-term GH therapy ^15^. Furthermore, treatment of adults with GHD is also associated with significant health care costs. In the UK, the average annual cost of GH treatment in adults is estimated to be around £3,350 per patient, with a life-long therapy cost of between £42,000 and £45,400 ^7^. Furthermore, the treatment regimen of daily self-injections can be inconvenient and burdensome for many patients ^14, 16^.

The current NICE guidance provides clear criteria for commencing GH treatment for adults with GHD. However, it but does not offer any recommendations on the optimal duration of GH treatment in adults or if and when discontinuation should be considered. Consequently, GH replacement therapy tends to be offered indefinitely even when patients fail to report any sustained benefits from this treatment. Given that GH treatment is associated with significant health care costs, it is imperative to clearly establish the clinical and cost-effectiveness of the therapy. A methodologically robust and well-designed large-scale multi-centre study will contribute this evidence. However, Medical Research Council guidelines suggest that feasibility studies are required to test the methodology and establish the specific parameters prior to any full large-scale multi-centre study ^17^. The strict criteria for prescribing GH to adults and the homogeneity of the UK population of adults with GHD, offers an ideal setting to undertake a robust study.

## Protocol

This is a sequential mixed-methods study which will be conducted in three phases:

- Phase 1: Survey of endocrine clinicians
- Phase 2: Observational feasibility cohort study
- Phase 3: Qualitative study

### Phase 1: Survey of endocrine clinicians

The aim of this phase is to understand the current practice of endocrine clinicians in the UK of offering GH treatment discontinuation in adults with GHD who have been prescribed long-term replacement therapy. It will also determine the willingness of endocrine clinicians in the UK to take part in a future large-scale multi-centre study.

***Methods***

A multiple-choice questionnaire has been developed drawing on current guidance, gaps in the evidence, as well as expert clinicians' opinion. The survey questions have been reviewed for content validity by expert clinicians who are involved in the study centre’s Growth Hormone Clinic and by members of the GAMBOL Patient and Public Advisory Group (PPAG). Formal endorsement will be sought from the Society for Endocrinology (SfE) to allow the survey to be sent to all endocrine clinicians (doctors and nurses) on the Society's contact list using Survey Monkey®. The questionnaire has been designed to ascertain the following:

- the number of adult patients treated with GH in different endocrine centres in the UK who have been on replacement therapy for more than 5 years;
- the current practice and criteria used by endocrine clinicians in offering a period of GH treatment discontinuation in adults;
- the interest of endocrine clinicians in taking part in future a large-scale multi-centre study on GH treatment discontinuation in adults.

***Survey administration***

Following approval by the SfE, the link to the Survey Monkey® online survey will be sent to all endocrine clinicians in the UK registered on the SfE membership database. Information will also be posted on the SfE website and on the endocrine nurses’ closed Facebook® forum. The survey is planned to be available for 8 weeks.

***Data collection and analysis***

Data will be collated from the survey and presented as frequencies and percentages. Descriptive data will be presented in themes when applicable.

***Presentation and dissemination***

Survey results will be presented initially at the study centre’s endocrine department’s monthly meeting. The results will also be presented at the Pituitary Foundation patient group meeting that takes place quarterly in Birmingham. An abstract will be submitted at the SfE congress highlighting the key findings from the survey.

### Phase 2: Observational feasibility cohort study

This phase will be conducted over 32 months to allow allow steady recruitment of adequate number of study participants, completion of outcome measures and evaluation and assessment of patients’ body composition (BMD [lumbar spine and both hips], muscle mass and fat mass [whole body] using DXA scan), at baseline and at two years. The 2-year interval for DXA scanning is necessary as reassessing patients’ BMD earlier would likely fail to capture any measurable significant changes ^15^.

***Primary aim***

To assess the feasibility of conducting a large-scale multi-centre study on discontinuation of long-term GH treatment in adult patients with GHD.

***Secondary aim***

To assess the impact of discontinuing long-term GH treatment on the metabolic profile, body composition and QoL in adult patients with GHD.

***Objectives***

- To assess the rate of patient recruitment, retention and withdrawal.
- To identify barriers for recruiting and retaining participants into the study.
- To assess the suitability and acceptability for patients of the frequency of follow-up and assessment.
- To assess the suitability and acceptability for patients of the QoL questionnaires.
- To assess the study protocol including recruitment, biochemical monitoring, body composition monitoring, and QoL assessment.
- To assess the safety of GH treatment discontinuation in adult patients with GHD.

***Study participants***

The study will recruit a total of three groups of adult patients with GHD (aged 25 years and over).

i. ***Group 1- Discontinuation group***: 20–25 patients who have been on GH therapy for 5 years or longer and voluntarily agree to discontinue their GH treatment and be monitored for two years.
ii. ***Group 2- Continuation group***: 20–25 patients who have been on GH therapy for 5 years or longer and voluntarily agree to take part in the study, where their GH treatment is continued and are monitored in the same way as the discontinuation group.
iii. ***Group 3- Continuation group following initial discontinuation***: patients who were initially part of the discontinuation group (Group 2) and decided to withdraw from the study, due to adverse effects of GH discontinuation, and restart their treatment. Patients monitoring will continue in the same way as the continuation group for a total of two years from the time they initially enrolled in the study.

***Study Visits***

Visit 1

- Patients attend endocrine outpatient clinic on the morning of the study visit after an overnight fast (fasted for at least 8 hours prior to study visit).
- Informed consent will be re-confirmed.
- Patients who do not consent to take part in the study will be referred back to the endocrine clinic to continue standard care monitoring.
- Following confirmation of consent, a standardised physical assessment, medical and medication histories will be completed to re-confirm inclusion/exclusion criteria.
- Study participants will undergo the following anthropometric assessments and all results will be recorded on the patient’s electronic health records: height; weight; BMI; blood pressure and, waist and hip circumferences. Height will be measured to the nearest centimetre (cm) using a wall-mounted meter. Weight will be measured in kilograms (kg) (to one decimal place) using an electronic scale. Waist circumference will be measured midway between the lower margin of the last palpable rib and the top of the iliac crest, and hip circumference at the level of the greater trochanters to the nearest cm. Blood pressure will be measured using an automated blood pressure monitor (Omron 705IT sphygmomanometer or equivalent). Blood pressure measurements will be performed at sitting position after a few minutes rest.
- Blood samples for research will be taken which include: insulin growth factor-1 (GF-1); high density lipoprotein (HDL); low density lipoprotein (LDL); total cholesterol; triglyceride; glycated haemoglobin A1c (HbA1c), and fasting glucose. For these purposes, blood samples will be drawn using one purple top EDTA tube (4mL x 1 = 4mL), one fluoride oxalate grey top tube (4mL x 1 = 4mL), one yellow top tube (6ml x 1 = 6mL) and 1 red top tube (6mL x1 = 6mL). A total of 4 blood tubes will be drawn with a total volume of 20mL.
- Study participants will complete three QoL assessment questionnaires which include: QoL-AGHDA (5 minutes); Treatment-Related Impact Measure Adult Growth Hormone Deficiency (TRIM-AGHD) (5 minutes) and, SF-36 (10 minutes).
- Study participants will undergo body composition assessment using DXA scan to measure BMD at the lumbar spine and both hips, as well as fat mass and muscle mass of the whole body. BMD and fat and muscle mass measurements will be completed in one DXA appointment.
- Appointment for study visit 2 (at 6 months [+/- 2 weeks]) will be arranged and confirmed.

Visits 2–4 (6-monthly visit) (between 08.00 AM – 12.00 Noon)

- Visits 2–4 will take place at month 6, 12 and 18 respectively (+/- 2 weeks).
- To allow some flexibility and to accommodate the study participant’s availability, each follow-up study visit can take place up to two weeks earlier or two weeks later than the planned scheduled visit.
- Study participants attend endocrine outpatient clinic on the morning of the study visit after an overnight fast (fasted for at least 8 hours prior to study visit).
- Re-confirm consent and eligibility criteria.
- A standardised physical assessment, medical and medication histories will be completed to assess for adverse events and to confirm each study participant’s suitability and willingness to continue with the study.
- Study participants will undergo the following anthropometric assessments and all results will be recorded on the patient’s electronic health records: height; weight; BMI; blood pressure and, waist and hip circumferences. Height will be measured to the nearest centimetre (cm) using a wall-mounted meter. Weight will be measured in kilograms (kg) (to one decimal place) using an electronic scale. Waist circumference will be measured midway between the lower margin of the last palpable rib and the top of the iliac crest, and hip circumference at the level of the greater trochanters to the nearest cm. Blood pressure will be measured using an automated blood pressure monitor (Omron 705IT sphygmomanometer or equivalent). Blood pressure measurements will be performed at sitting position after a few minutes rest.
- Blood samples for research will be taken which include: IGF-1; HDL; LDL; total cholesterol; triglyceride; HbA1c, and fasting glucose. For these purposes, blood samples will be drawn using one purple top EDTA tube (4mL x 1 = 4mL), one fluoride oxalate grey top tube (4mL x 1 = 4mL), one yellow top tube (6ml x 1 = 6mL) and 1 red top tube (6mL x1 = 6mL). A total of 4 blood tubes will be drawn with a total volume of 20mL.
- Study participants will complete three QoL assessment questionnaires which include: QoL-AGHDA (5 minutes); TRIM-AGHD (5 minutes) and, SF-36 (10 minutes).
- Appointment for study visit 5 will be arranged and confirmed.

Visit 5 (month 24 [+/- 2 weeks]) (between 08.00 AM – 12.00 Noon)

- To allow some flexibility and to accommodate the study participant’s availability, as well the availability of slots for DXA scan appointment, this final study visit can take place up to two weeks earlier or two weeks later than the planned scheduled visit.
- Study participants attend endocrine outpatient clinic on the morning of the study visit after an overnight fast (fasted for at least 8 hours prior to study visit).
- Re-confirm consent and eligibility criteria.
- A standardised physical assessment, medical and medication histories will be completed to assess for adverse events.
- Study participant will undergo the following anthropometric assessments and all results will be recorded on the patient’s electronic health records: height; weight; BMI; blood pressure and, waist and hip circumferences. Height will be measured to the nearest centimetre (cm) using a wall-mounted meter. Weight will be measured in kilograms (kg) (to one decimal place) using an electronic scale. Waist circumference will be measured midway between the lower margin of the last palpable rib and the top of the iliac crest, and hip circumference at the level of the greater trochanters to the nearest cm. Blood pressure will be measured using an automated blood pressure monitor (Omron 705IT sphygmomanometer or equivalent). Blood pressure measurements will be performed at sitting position after a few minutes rest.
- Blood samples for research will be taken which include: IGF-1; HDL; LDL; total cholesterol; triglyceride; HbA1c, and fasting glucose. For these purposes, blood samples will be drawn using one purple top EDTA tube (4mL x 1 = 4mL), one fluoride oxalate grey top tube (4mL x 1 = 4mL), one yellow top tube (6ml x 1 = 6mL) and 1 red top tube (6mL x1 = 6mL). A total of 4 blood tubes will be drawn with a total volume of 20mL.
- Study participants will complete three QoL assessment questionnaires which include: QoL-AGHDA (5 minutes); TRIM-AGHD (5 minutes) and, SF-36 (10 minutes).
- Study participants will undergo body composition assessment using DXA scan to measure BMD at the lumbar spine and both hips, as well as fat mass and muscle mass of the whole body. BMD and fat and muscle mass measurements will be completed in one DXA appointment.
- This will be the end of phase two study visits.
- Participants who are eligible to take part in the phase 3 of the study will be approached. Interested participants will be provided with patient information sheet and will have a minimum of 72 hours to decide if they wish to participate. Potential participants will be contacted by phone after 72 hours to confirm their willingness to take part in the phase 3 of the study and patients who confirmed their willingness for this will be booked an appointment for either a face-to-face, a telephone or a virtual (via Microsoft Teams) interview (according to participant’s preference). Any patient who wishes to have more time to consider the study will be given a further 72 hours.

***Data collection and analysis***

Data will be collected and stored in the study centre’s secured and password protected server. A GAMBOL study data sheet will be generated using ReDCAP® which is password protected. All data will be pseudo-anonymised. Data analysis will involve descriptive statistics and presented as means ± standard deviation (SD) for continuous variables that are normally distributed, and as medians with interquartile (25th and 75th percentile) range for continuous variables that are skewed. Between-group differences will be analysed using independent samples t test and analysis of covariance (ANCOVA) test. Categorical variables will be presented as frequencies and percentages. Statistician from UHB has agreed to provide support for this study.

### Phase 3: Qualitative study (Interview)

#### The aim of this part of the study is to explore patients’ experiences of participating in the study and understand any barriers or facilitators to retention and non-retention.

***Objectives***

- To identify and explore the reasons for patients choosing to participate in the study and their selection of taking part in the discontinuation or continuation group.
- To capture experiences of study participation.
- To identify the factors that facilitated retention of participants in the study.
- To identify reasons why participants choose to withdraw from the study.
- To explore participants’ views on the use of randomisation in future large-scale multi-centre study.

***Study Design: Descriptive qualitative methodology***

Using purposive sampling, 5–8 participants who completed the study, and similar number of participants who withdrew from phase 2 of the study, will be invited to participate in this phase. On completion of (or withdrawal from) phase 2 study, all participants will be given a PIS explaining this phase. Informed written consent will be taken and recorded. This will be reaffirmed when participants attend for their interview.

Participants will be offered a choice between face-to-face, telephone or virtual (via Microsoft Teams) interviews. These options were informed by advice from members of the PPAG, advice from researchers who had explored or worked on similar themes, literature suggesting that decliners would be reluctant to take part in face-to-face interviews ^16^, evidence that well-planned telephone interviews can gather similarly rich data as face-to-face interviews ^16^, and learning from coronavirus pandemic. Interviewing will continue until saturation is reached, estimated around 12 informants ^17^.

A topic guide will be developed in consultation with the members of the PPAG and supervisory team. It is envisaged the interviews will explore the experience of participating in the study, the factors that influence the participants to complete or withdraw and the feelings of the participants regarding the use of randomisation in any future large-scale study. All interviews will be digitally recorded on a password protected device or software (e.g. Microsoft Teams) and transcribed verbatim by the chief investigator (subject to confidentiality and security of data transfer arrangements) in compliance with study centre’s data management policies. These data will be stored in the study centre’s secured and password protected server.

All transcribed interview data will be analysed using thematic analysis ^18, 19^. Transcripts will be imported in NVivo to facilitate data management. After familiarisation, reading and re-reading each transcript against sound file, coding will be undertaken. Sections of text will be named producing a list of codes. In collaboration with my supervisors, a thematic schema will be formed, which may initially undergo numerous stages of refinement, and ultimately produce an analysis of participants’ accounts.

### Study setting

Participants of the GAMBOL study (Phase 2 and Phase 3) will be identified, recruited, and assessed from the two endocrine outpatient clinics of the University Hospitals Birmingham NHS Foundation Trust listed below:

- Queen Elizabeth Hospital Birmingham
- Birmingham Heartlands Hospital

### Eligibility criteria: Phase 2

Inclusion criteria:

- Patients aged 25 years and over with GHD.
- GHD previously confirmed by a validated test as per guideline ^7^.
- On GH replacement therapy for 5 years or longer.
- Able to provide informed written consent.

Exclusion criteria:

- Patients with poorly controlled diabetes (defined as having an recent HbA1c of >7%), poorly controlled hyperlipidaemia and severe cardiovascular disease.
- Patients receiving treatment for low BMD or deemed to be requiring treatment for low BMD during baseline screening.
- Patients who are unable to provide informed written consent.

### Eligibility criteria: Phase 3

Participants who completed phase 2 of the study and participants who withdrew (at any point) from phase 2 are eligible to take part in the phase 3 of the study.

### Sampling

***Size of sample***

As this is a feasibility study, a formal power calculation is not required ^20^. A maximum of 25 patients on each group (50 in total) will be recruited in the phase 2 which is at the upper end of the numbers recommended for pilot/feasibility studies ^21^. 5-8 participants who completed the phase 2 , and similar number of participants who withdrew from phase 2, will be invited to participate in the phase 3 of the study.

***Sampling technique and recruitment***

Phase 2 (Observational Feasibility Cohort Study): purposive case sampling will be used to recruit study participants. Eligible patients will be identified from endocrine outpatient clinic lists and from the endocrine database of patients on growth hormone treatment. Patients who are willing to take part in the study will be given the choice of taking part in either the discontinuation group or the continuation group. Patients withdrawing from the discontinuation group will be invited to continue on the study for on-going monitoring.

Phase 3 (Qualitative Study: Interview): purposive sampling will be used to recruit study participant. Participants will be recruited from those taking part in the phase 2 of the GAMBOL study. Participants will be approached at the point of completing the phase 2 (study visit 5), whereas those who are withdrawing from phase 2 of the study will be approached at the point of withdrawal.

### Recruitment

***Sample identification (Phase 2)***

Participants will be identified from the following three primary sources:

i. By the clinical team through the outpatient endocrine clinic attendance at the Queen Elizabeth Hospital Birmingham and Birmingham Heartlands Hospital during patients’ routine follow-up appointment;
ii. By the chief investigator through the UHB endocrine database of patients on GH treatment, and
iii. By the chief investigator from patients responding to advertisement posted on the UHB Trust research website and the Pituitary Foundation’s website and social media platforms.

***Sample identification (Phase 3)***

Two groups of participants will be recruited from those taking part in the phase 2 of the GAMBOL study. Those who completed the phase 2 will be approached at the point of completion (study visit 5), and those who are withdrawing from phase 2 will be approached at the point of withdrawal.

## Dissemination (Phase 2 and Phase 3)

The results of this study will be submitted as abstracts at national and international endocrine conferences and for publication in peer-reviewed journals. Results will be also disseminated to participants, with the support of the GAMBOL Study PPAG members, using a participant newsletter and through the Pituitary Foundation local and national meetings.

## Consent

Written and verbal consent will be obtained from all participants involved in the study by the chief investigator. A copy of the signed informed consent form (ICF) will be filed in the medical notes, and the original will be placed in the Investigator Site File (ISF). Patients who decline to take part in the study will be reassured that their care will continue in the endocrine clinic as planned.

## Withdrawing from the study

Any research participant can withdraw from the study (phase 2 or phase 3) at any point. Participants on the GH treatment discontinuation group who choose to withdraw from the study due to side effects and wish to restart GH treatment, will be invited to continue on the study, as part of study group 3, for on-going monitoring whilst they restart their treatment. This will allow monitoring if their symptoms improve following re-introduction of GH treatment. Only participants who consented will continue on the study. 

Withdrawal can be made in person during the follow-up study visit, by phone or by email without the need to explain the reason for withdrawal. Withdrawal from the study will be documented on the patient’s clinical notes and patient’s GP, as well as the endocrine consultant, will be informed in writing.

## Ethical and Regulatory Considerations

**Ethics Reference Number:** HRA and Health and Care Research Wales (HCRW) Approval (23/WA/0105).

**Approval date:** 17 April 2023

REC approval has been sought for the phase 2 and phase 3 of the GAMBOL study. The study will be performed in accordance with the recommendations from the ‘World Medical Association Declaration of Helsinki’, adopted by the 18th World Medical Association (WMA) General Assembly, Helsinki, Finland, 1964, amended by the 48th WMA General Assembly, Somerset West, Republic of South Africa, 199

The study will also be conducted in accordance with the Research Governance Framework for Health and Social Care, the applicable UK Statutory Instruments, (which include the General Data Protection Regulation and Data Protection Act 2018 and Human Tissue Act 2008) and the principles of Good Clinical Practice (GCP).

Risk will be assessed and managed at each stage of the ethical review process. The overall risk to the study participants is low as no serious and long-lasting effects are expected.

### Assessment and management of risk

Risk will be assessed and managed at each stage of the ethical review process. UHB, as sponsor, has comprehensively reviewed the study protocol, before submission to the Research Ethics Committee, and will monitor the conduct of the study. UHB’s Research and Development (R&D) department has also reviewed and approved the study. The overall risk to the study participants is low as no serious and long-lasting effects are expected (as described in detail below).

The following risks are anticipated in the study:

a. Participants will undergo blood test procedure at each study visit. The site of venepuncture might bleed or bruise. These will be minimised by the use of good clinical practice and sterile techniques. To avoid subjecting participants to multiple invasive phlebotomy procedures, all baseline biochemical measures will be taken after eligibility is confirmed. The volume of blood to be taken at each visit would be maximum of 20 ml. It is safe losing this amount of blood each attempt at venepuncture at each visit and there are no adverse effects to expect from this procedure.
b. The participants will arrive fasted for their study visit but will be allowed to eat as soon as blood samples have been taken.
c. If any of the routine baseline bloods are outside the normal range, the patient, their endocrinologist and their GP will be informed and further advice will be provided.
d. Participants will be exposed to ionising radiation by undergoing DXA scan. DXA scan is used routinely in clinical practice to assess baseline bone density and body composition in adult patients with GHD. However, some study participants will receive additional radiation burden from the repeat scan at 24 months, which is above that anticipated in routine clinical care. In clinical practice, only those patients with abnormal baseline DXA scan undergo a repeat scan after 2–3 years to assess any further deterioration or response to treatment (if bone metabolism medication is started). In this study, all study participants will have a repeat DXA scan regardless of their baseline scan result. The risk of harm from the additional radiation burden is low and is justified to provide objective radiological assessment during the study. The scan will be performed using the GE Lunar iDXA scanner. The amount of ionising radiation that each participant will be exposed to during each DXA scan procedure is around 0.03 milliSieverts (mSv) (lumbar spine= 0.013 mSv; both hips= 0.009 mSv; whole body= 0.008 mSv) and equivalent to around 5 days of background radiation in the UK (Damilakis *et al.* 2010. Eur Radiol (2010) 20 (11): 2707–2714). Each participant will undergo DXA scan procedure twice (at baseline and at 24 months) during the duration of the study. The total amount of ionising radiation from the two DXA scans is 0.06 mSv which is equivalent to 10 days of natural background radiation in the UK. The interval between baseline and repeat DXA scan at 24 months is in line with standard practice as per the sponsor's guideline for DXA scan. Ionising radiation can cause cancer which manifests itself after many years or decades. The risk of developing cancer as a consequence of taking part in this study is 0.0003%, which is low. For comparison, the natural lifetime cancer incidence in the general population is about 50%. DXA scan will be performed by qualified DXA scan practitioner who will be practicing in accordance with sponsor’s clinical guideline and the Ionising Radiation Regulations 2017.
e. Participants will be asked to complete three Quality of Life questionnaires at each study visit and a final end of study questionnaire. This might be inconvenient to them in terms of time. Questionnaires validated for adults with GHD have been selected which contains minimal amount of data that meet the research requirement and the time needed to complete these questionnaires have been factored in at each study visit.
f. Participants in the discontinuation group will be asked to stop their GH treatment for 2 years. During the period of GH treatment discontinuation, some participants may experience symptoms of GHD. Patients reporting symptoms of GHD or any other adverse events will be assessed promptly and those who wish to restart their GH treatment will be re-started promptly, withdraw from the study and transfer to standard care. The risks associated with GH treatment discontinuation will be minimised as follows:
  • low mood- participants will be advised at the start of the study, supported and reinforced by the PIS and during each study visit, to contact the study team if they have concern regarding low mood. Prompt initiation of GH treatment will be offered to participants who wish to restart their treatment;
  • poor general well-being- participants will be advised at the start of the study, supported and reinforced by the PIS and during each study visit, to contact the study team if they have concern regarding poor general well-being. Prompt initiation of GH treatment will be offered to participants who wish to restart their treatment;
  • low energy levels- participants will be advised at the start of the study, supported and reinforced by the PIS and during each study visit, to contact the study team if they have concern regarding low energy level. Prompt initiation of GH treatment will be offered to participants who wish to restart their treatment ;
  • reduced bone mineral density (BMD)- BMD will be measured at baseline and at 24-months. Existing national guideline (NOGG, 2017) on managing low BMD will be followed and patients who require further assessment will be referred to their GPs. Patients who require active intervention for low BMD (following baseline assessment) will be excluded from the study. Prompt initiation of GH treatment will be offered to participants who wish to restart their treatment;
  • increased body fat- body fat mass will be measured at baseline and at 24-months. The importance of active lifestyle measures (e.g., regular exercise and healthy-balanced diet) will be reinforced to all participants at the start of the study, supported by patient information sheet and during each study visit. Patients who require active intervention, following baseline assessment, will be excluded from the study. Prompt initiation of GH treatment will be offered to participants who wish to restart their treatment;
  • increased cholesterol levels- cholesterol levels will be measured at baseline and every 6 months for 2 years. Existing national guideline on managing high cholesterol level will be followed and patients who require further assessment will be referred to their GPs. Patients who require active intervention for high cholesterol levels, following baseline assessment, will be excluded from the study. Prompt initiation of GH treatment will be offered to participants who wish to restart their treatment;

g. During the study period, each participant will be required to attend a total of 5 study visits for adverse events assessment, blood sampling, completion of questionnaires and measurements of body composition. The total number of planned study visits has been limited to the minimum requirement for the study which will ensure safety and appropriate follow-up of each participant. Each study visit will take place at the participants' usual site of care and standard travel cost will be reimbursed to reduce the financial burden of travel.

## Safety reporting

### Reporting requirements

The collection and reporting of Adverse Events (AEs) will be in accordance with the Research Governance Framework for Health and Social Care and the requirements of the Health Research Authority. The chief investigators will assess the seriousness and causality (relatedness) of all AEs experienced by any study participant with reference to the protocol. Participants experiencing any AE will also be referred to their endocrinology consultant for further assessment and management if required.

### Adverse events

All medical occurrences, including symptoms of GHD and out of range laboratory and BMD values, which meet the definition of an AE will be reported. All AEs occurring during the study observed by the investigator or reported by the participant, whether attributed to study or not, will be recorded on the patient’s medical notes. The following information will be recorded: description, date of onset and end date, severity, assessment of relatedness to study, other suspect device and action taken. Follow-up information should be provided, as necessary.

AEs considered related to the study as judged by patient’s endocrinology consultant will be monitored until resolution, or until the event is considered stable. All related AEs that result in a participant’s withdrawal from the study or are present at the end of the study, will be followed up until a satisfactory resolution occurs. Any participant with a serious adverse event will undergo assessment by their endocrinology consultant and be given appropriate care under medical supervision until symptoms cease or the condition becomes stable. The severity of events will be assessed on the following scale: 1 = mild, 2 = moderate, 3 = severe. The relationship of AEs to the study will be assessed by participant’s endocrinology consultant.

Each severe (serious) AEs (SAEs) will be reviewed by the chief investigator and the participant’s endocrinology consultant who will classify the AEs into one of the following two categories: ****Suspected Unexpected Serious Adverse Reaction (SUSAR) (which may or may not be fatal or life threatening) and Suspected Serious Adverse Reaction (SSAR). Rare but recognised AEs will not be considered as ‘unexpected****’.

### Reporting period

For each individual participant, details of all AEs will be documented and reported for the duration of participation in the study, i.e., from the point of consent up until the last follow-up visit. The investigator will report all SAEs to the sponsor immediately upon knowledge of the event. This will be done by informing the research and development office that a serious adverse event has occurred verbally or in writing which will be followed by a detailed written report on the event. This report will be kept in the Investigator Site File. The chief investigator and the participant’s endocrinology consultant will assess the seriousness and severity of AEs on behalf of the sponsor.

The sponsor will ensure that all relevant information about a SUSAR which occurs during the course of the clinical study and is fatal or life threatening is reported as soon as possible to the relevant Ethics Committee no more than seven calendar days after the sponsor was first made aware of the event. Any additional relevant information will be sent as soon as available.

### Reporting procedure

The chief investigator is responsible for onward reporting of any AEs. A fully documented audit trail will be maintained at the study site. A report of all SAEs (including non-SUSAR events) will be submitted annually to the main Research Ethics Committee (REC).

AEs will be documented in the participant clinical notes. The chief investigator will evaluate any AEs reported and, if appropriate, will report further to the relevant authorities (Health Research Authority/Research Ethics Committee, UHB). The chief investigator will report a minimal dataset of all individual events categorised as a fatal or life threatening SUSAR to the Health Research Authority/Research Ethics Committee within 7 days. Detailed follow-up information will be provided within an additional 8 days.

For adverse events categorised as SUSARs, these will be reported within 15 days. A copy is also sent to the UHB Research Governance Team at the time of sending the SUSAR report. The Health Research Authority/Research Ethics Committee will be notified immediately if a significant safety issue is identified during the study, with the UHB Research Governance Team also notified at the same time.

Any study participants can contact the chief investigator by email ( Sherwin.criseno@uhb.nhs.uk) or by phone (0121-371-6950) at any point during the study (Monday to Friday, between 08.00H – 16.00H) to report any adverse events or concerns about taking part/continuing with the study.

## Amendments

Any amendments (substantial or non-substantial) will be approved by UHB R&D. The chief investigator will submit a notice of amendments to the REC for consideration and will report to the Health Research Authority/REC using the appropriate processes. Amendment history will be tracked at the end of the protocol (section 11.3), reporting the amendment number, protocol version number, date amended, and approval issued, author(s) of changes and details of changes made.

## Trial registration

The trial is registered (04.08.2023) with the National Library of Medicine ClinicalTrials.gov, registration number NCT05979480.

Protocol version: GAMBOL_STUDY_PROTOCOL-(Version1-2_20 March 2025)

## Trial status

Recruitment to this study started in July 20 2023 and completed in May 31 2024. Study participants are expected to complete the phase 2 of the study in May 31 2026. Phase 3 of the study is expected to be completed in June 30 2026. Analysis of the data from phase 2 of the study will commence 4 weeks after completion of the final study visit of the last study participant. Analysis of data from phase 3 of the study will commence 4 weeks after completion of the interview of the last study participant. Database will be cleaned and locked after the data analysis is completed. The current protocol version is Version1-2_20 March 2025.

## Trial oversight

### Protocol compliance

***Site set-up and initiation***

Prior to commencing recruitment, all members of the GAMBOL study team (chief investigator and primary supervisors) will have completed GCP training or refresher training within the last two years. An Investigator File will be created to contain essential documentation, instructions, and other documentation required for the conduct of the study.

### Oversight

Oversight of the study will be provided by the chief investigator who will report any relevant issues arising from the conduct of the study to the Health Research Authority/Research Ethics Committee and the sponsor (UHB).

### Audit and inspection

The study will be monitored and/or audited by University Hospitals Birmingham NHS Foundation Trust under their remit as sponsor and other regulatory bodies to ensure adherence to Good Clinical Practice and the UK Health Policy Framework for Health and Social Care.

Monitoring of study data shall include confirmation of informed consent; source data verification; data storage and data transfer procedures; local quality control checks and procedures, back-up and disaster recovery of any local databases and validation of data manipulation. The study coordinator or where required, a nominated designee of the sponsor, shall carry out monitoring of study data as an on-going activity.

Once the first study participant is enrolled into the study and their first visit completed, the data and adherence to protocol will be monitored by the sponsor’s quality assurance (QA) team. Monitoring of study participants by the sponsor’s QA team will then occur at random intervals throughout the study which could be in the form of self-monitoring tools being supplied.

Study conduct will be subject to systems audit of the study record for: inclusion of essential documents; permissions to conduct the study; study delegation log; CVs of study members and training received; local document control procedures; consent procedures and recruitment logs; adherence to procedures defined in the protocol (e.g. inclusion / exclusion criteria, timeliness of visits), and accountability of study materials. This will be led by the chief investigator and reported back to the sponsor.

Entries on the data sheet will be verified by inspection against the source data. A sample of participants’ data (approximately 10%) will be checked on a regular basis for verification of all entries made. In addition, the subsequent capture of the data on the study database will be checked. Where corrections are required these will carry a full audit trail and justification.

Study data and evidence of monitoring and systems audits will be made available for inspection by the regulatory authority where applicable as required. The data and adherence to protocol will be monitored by the sponsor’s Q&A team at the earliest opportunity.

### Protocol deviations, non-compliances, and breaches

Accidental protocol deviations can happen at any time. They will be adequately documented on the relevant forms by the chief investigator and reported immediately to the Sponsor. Deviations from the protocol which are found to frequently recur are not acceptable and will be dealt with immediate action and could potentially be classified as a serious breach. Any major problems identified during monitoring may be reported to the Health Research Authority/Research Ethics Committee. This includes reporting serious breaches of GCP and/or the study protocol. A copy of the report will be sent to the UHB R&D Compliance team at the time of reporting to the REC and/or relevant regulatory bodies.

### Data protection and patient confidentiality

As per the Confidentiality- NHS Code of Practice 2003, data will be collected and kept confidential according to the Data Protection Act 2018, GDPR and Caldicott Principles. Minimum data will be collected to enable safe participation in the study. Data will be collected and stored in the NHS Trust's secured and password protected server. Access to the NHS computer server is via an authorised login and password only. Data collected during each study visit and questionnaires will be pseudonymised to include unique identifier, date of birth and sex only. Blood sample and bone density scan forms will have pseudonymised information including unique identifier, date of birth and sex to enable correct biochemical and radiological reference ranges to be used.

Patient's main clinical care team will have usual access to the participant's personal data. In addition, the GAMBOL study team will have access to participants’ personal data for the purposes of the study only. This is to allow safety monitoring and organisation of required follow up appointments. NHS Trust laboratory and imaging departments will receive pseudo-anonymised data for the purposes of analysis of samples and provision of results. The sponsor may require access to participants’ personal data for the purposes of audit and inspection. This will be explained in the participant information sheet and written consent will be sought from participants.

Data provided by the participants for the study will be stored in paper and electronic forms in the study centre' NHS computers and premises. The chief investigator and members of the GAMBOL study team will have access to medical records in order to document the patients' participation in the study and any interventions carried out during the course of the research. A manual paper record of participant addresses, postcodes and telephone numbers will be maintained in the investigator site file (ISF) to enable correspondence or contact with participants during the course of the study. Contact will be related to the patient's participation in the current research project only. The Trust's laboratory and imaging department will store participants' data on NHS servers. The ISF will be kept in a locked filing cabinet within a lockable office. Access to the ISF will be restricted to the GAMBOL study team only. No confidential information will be asked from the participants unless consent is given. Questionnaire data will be securely stored in the NHS server which is password-protect and encrypted in situ. No data will be transferred out of University Hospitals Birmingham NHS Foundation Trust. Data will be analysed by the GAMBOL study team and the UHB Trust statistician from the Trust password protected server.

### Patient and Public Involvement

Patient and public involvement has been integral from the inception through to the design of the study. Expert advice was received from the study centre’s PPI lead who also provided guidance in ensuring that the approach was aligned with the six UK standards for public involvement ( https://sites.google.com/nihr.ac.uk/pi-standards/home). The study centre’s PPI lead, who fully supports this study, reviewed various iterations of the project proposal and guided the PPI activities. The Pituitary Foundation (PF) is a national patient support group for patients with pituitary disorders and their families.

In January 2020, at the annual UHB Pituitary Open Morning meeting, the GAMBOL PPAG was formally established consisting of three adult patients with GHD and two family members. Since forming the GAMBOL PPAG, members have reviewed the study protocol on two occasions. This collaborative and inclusive approach will continue through the lifetime of the study, to ensure the research is appropriate and acceptable. They will inform study design, data collection, interpretation and dissemination.

## Conclusions/Discussion

Many questions on the effectiveness of long-term treatment with GH in adults remain unanswered including its optimal duration and the consequences of treatment discontinuation. Currently, much of the evidence on safety-related issues associated with long-term GH therapy comes from uncontrolled retrospective, and observational studies, which are methodologically inferior compared with RCTs ^1, 6^. A critical assessment of data from limited RCTs did not show consistent beneficial effects of GH therapy in adult patients with GHD compared with placebo ^6^.

In 2016, several international endocrine societies collectively highlighted the need for carefully designed and rigorously conducted cohort studies to monitor the safety of long-term GH therapy ^22^. Furthermore, treatment of adults with GHD is also associated with significant health care costs. In the UK, the average annual cost of GH treatment in adults is estimated to be around £3,350 per patient, with a life-long therapy cost of between £42,000 and £45,400 ^7^. Furthermore, the treatment regimen of daily self-injections can be inconvenient and burdensome for many patients ^14, 23^.

The current NICE guidance provides clear criteria for commencing GH treatment for adults with GHD. However, it does not offer any recommendations on the optimal duration of GH treatment in adults or if and when discontinuation should be considered. Consequently, GH replacement therapy tends to be offered indefinitely even when patients fail to report any sustained benefits from this treatment. Given that GH treatment is associated with significant health care costs, it is imperative to clearly establish the clinical and cost-effectiveness of this therapy.

A methodologically robust and well-designed large-scale multi-centre study will contribute this evidence. However, Medical Research Council guidelines suggest that feasibility studies are required to test the methodology and establish the specific parameters prior to any full large-scale multi-centre study ^24^. The strict criteria for prescribing GH to adults and the homogeneity of the UK population of adults with GHD offer an ideal setting to undertake a robust study.

## Data Availability

No underlying data available at this stage of the study. GAMBOL study patient information sheets, informed consent forms and SPIRIT checklist are available on Figshare: Criseno, Sherwin (2025). GAMBOL STUDY Patient Information Sheets and Consent Forms. figshare. Preprint. https://doi.org/10.6084/m9.figshare.30112093.v1 ^25^. Data are available under the terms of the Creative Commons Attribution 4.0 International license (CC-BY 4.0).
