# Supplementary material for: The effects of Growth hormone treatment discontinuation in Adults on Metabolic profile, Body composition and quality Of Life (GAMBOL Study)
Source: NIHR Open Res. 2026 Aug 19;5:83. Originally published 2025 Sep 19. [Version 2] doi: 10.3310/nihropenres.14030.2 (PMC12624266; doi:10.3310/nihropenres.14030.2)
Supplement: Peer review history

# Reviewer report: Reviewer response for version 1

**DOI:** 10.3310/nihropenres.15259.r37466

**Published:** 2025-11-17

**Authors:** Rob Murray

**Affiliations:** 1 Leeds Teaching Hospital NHS Trust, Leeds, UK

Criseno et al outline an interesting protocol to quantify the impact of discontinuation of growth hormone replacement on quality of life, body composition and metabolic parameters. The study opened in July 2023, completed recruitment in May 2024 and therefore is currently over half the way through phase 2.

 Comments:

 Following the “Plain Language Summary” only ‘phase 1 is qualified. The plain language summaries of phase 2 and phase 3 appear to be missing.

 How would analysis of data from phase 1 account for variable experience of Specialists completing the questionnaire? For example a specialist managing several hundred patients with GH replacement with that of a specialist managing a handful of patients?

 Is LDL-Cholesterol directly measured, or calculated? If the latter this should be made clear.

 Phase 2, Study participants, Group 3; On the third line the authors refer to “…the discontinuation group (Group 2) and decided…”. I believe this should be Group 1?

 The methodology for body composition measures, and blood samples is repeated for visit 1, visits 2-4 and visit 5. The details could be included at visit 1, and the methodology shortened for the additional visits.

 For Phase 3 would it be interesting to invite a group of patients who did not wish to enter the Phase 2 study on either Group 1 or 2?

 For the inclusion criteria, would additional hormone replacement therapies need to be stable for a period of time before recruitment? Additionally, changes in other hormone replacements can affect the variables being measured over the two years follow-up. How would this be monitored and dosage adjustments undertaken? For exclusion criteria should weight loss drugs be included given the widespread usage of this from private prescribers?

 For Group 3 in the Phase 2 study, would the patients undergo a further visit/assessment at the time of enrolment in to the Group 3 cohort, potentially including a further DXA assessment?

 There are a number of typographical errors which require addressing

Is the study design appropriate for the research question?

Yes

Is the rationale for, and objectives of, the study clearly described?

Yes

Are sufficient details of the methods provided to allow replication by others?

Yes

Are the datasets clearly presented in a useable and accessible format?

Yes

Reviewer Expertise:

Adult Endocrinology (Pituitary)

I confirm that I have read this submission and believe that I have an appropriate level of expertise to confirm that it is of an acceptable scientific standard, however I have significant reservations, as outlined above.

# Response

**Published:** 2026-08-06

**Authors:** Sherwin Criseno

**Affiliations:** Endocrinology and Metabolism, University Hospitals Birmingham NHS Foundation Trust, Birmingham, England, UK

Thank you Prof. Murray for your comments which we have considered.

 Comment 1

*Following the Plain Language Summary only Phase 1 is qualified. The plain language summaries of Phase 2 and Phase 3 appear to be missing.*

**We thank the reviewer for highlighting this point. The descriptions of Phases 2 and 3 are included within the Plain Language Summary; however, we agree that the formatting may not have made this sufficiently clear. We have revised the section to ensure that all three phases are clearly presented and consistently described for ease of understanding by patients and members of the public.**

 Comment 2

*How would analysis of data from phase 1 account for variable experience of specialists completing the questionnaire?*

**We thank the reviewer for this important observation. The primary purpose of Phase 1 is to obtain a broad understanding of current UK clinical practice rather than estimate prevalence or generate weighted national estimates. We have clarified that respondents will be asked to report the approximate number of adults with GHD managed within their service. This information will be used descriptively to contextualise responses and explore potential differences according to centre size and level of experience where appropriate.**

 Comment 3

*Is LDL-cholesterol directly measured, or calculated?*

**We thank the reviewer for highlighting this point. LDL cholesterol will be calculated using the standard laboratory methodology. This has now been clarified in the protocol.**

 Comment 4

*Group 3 refers to the discontinuation group (Group 2). Should this be Group 1?*

**We thank the reviewer for identifying this typographical error. The discontinuation group is Group 1 and the protocol has been corrected accordingly.**

 Comment 5

*Methodology for body composition measures and blood sampling is repeated across visits.*

**We agree with the reviewer. To improve readability and reduce duplication, the detailed methodology for anthropometric measurements, blood sampling and DXA assessment has been described at Visit 1, with subsequent visits referring back to the same methodology where appropriate.**

 Comment 6

*Would it be interesting to invite patients who declined participation in Phase 2?*

**We agree that the perspectives of patients who decline participation could provide valuable insights into barriers to recruitment and trial acceptability. However, the primary objective of Phase 3 is to explore experiences of participation, retention and withdrawal from the feasibility study. Given the scope of the current project and available resources, interviews are restricted to individuals who enrolled in Phase 2. We acknowledge that exploring the views of trial decliners would be an important area for future research.**

 Comment 7

*Should other hormone replacement therapies be stable before recruitment? How will changes be monitored? Should weight-loss drugs be excluded?*

**We thank the reviewer for these valuable suggestions. We agree that changes in other hormone replacement therapies could potentially influence study outcomes. We have therefore clarified that participants should be receiving stable replacement doses where clinically appropriate prior to enrolment, and that all adjustments to concomitant hormone therapies during follow-up will be recorded and considered during data analysis.**

 Comment 8

*Should Group 3 undergo an additional assessment or DXA scan at the time of transfer?*

**We thank the reviewer for this suggestion. Patients transferring to Group 3 will continue study follow-up according to the existing visit schedule. We considered performing an additional assessment at the time of transfer; however, this would increase participant burden and radiation exposure without directly contributing to the primary feasibility objectives. The timing of treatment re-initiation, reasons for withdrawal, symptoms and relevant clinical outcomes will be recorded as part of ongoing monitoring.**

 Comment 9

*There are a number of typographical errors which require addressing.*

 Comment 1

*Following the Plain Language Summary only Phase 1 is qualified. The plain language summaries of Phase 2 and Phase 3 appear to be missing.*

**We thank the reviewer for highlighting this point. The descriptions of Phases 2 and 3 are included within the Plain Language Summary; however, we agree that the formatting may not have made this sufficiently clear. We have revised the section to ensure that all three phases are clearly presented and consistently described for ease of understanding by patients and members of the public.**

 Comment 2

**Reviewer comment:**

*How would analysis of data from phase 1 account for variable experience of specialists completing the questionnaire?*

**We thank the reviewer for this important observation. The primary purpose of Phase 1 is to obtain a broad understanding of current UK clinical practice rather than estimate prevalence or generate weighted national estimates. We have clarified that respondents will be asked to report the approximate number of adults with GHD managed within their service. This information will be used descriptively to contextualise responses and explore potential differences according to centre size and level of experience where appropriate.**

 Comment 3

*Is LDL-cholesterol directly measured, or calculated?*

**We thank the reviewer for highlighting this point. LDL cholesterol will be calculated using the standard laboratory methodology. This has now been clarified in the protocol.**

 Comment 4

*Group 3 refers to the discontinuation group (Group 2). Should this be Group 1?*

**We thank the reviewer for identifying this typographical error. The discontinuation group is Group 1 and the protocol has been corrected accordingly.**

 Comment 5

*Methodology for body composition measures and blood sampling is repeated across visits.*

 We agree with the reviewer. To improve readability and reduce duplication, the detailed methodology for anthropometric measurements, blood sampling and DXA assessment has been described at Visit 1, with subsequent visits referring back to the same methodology where appropriate.

 Comment 6

*Would it be interesting to invite patients who declined participation in Phase 2?*

**We agree that the perspectives of patients who decline participation could provide valuable insights into barriers to recruitment and trial acceptability. However, the primary objective of Phase 3 is to explore experiences of participation, retention and withdrawal from the feasibility study. Given the scope of the current project and available resources, interviews are restricted to individuals who enrolled in Phase 2. We acknowledge that exploring the views of trial decliners would be an important area for future research.**

 Comment 7

*Should other hormone replacement therapies be stable before recruitment? How will changes be monitored? Should weight-loss drugs be excluded?*

**We thank the reviewer for these valuable suggestions. We agree that changes in other hormone replacement therapies could potentially influence study outcomes. We have therefore clarified that participants should be receiving stable replacement doses where clinically appropriate prior to enrolment, and that all adjustments to concomitant hormone therapies during follow-up will be recorded and considered during data analysis.**

 Comment 8

*Should Group 3 undergo an additional assessment or DXA scan at the time of transfer?*

**We thank the reviewer for this suggestion. Patients transferring to Group 3 will continue study follow-up according to the existing visit schedule. We considered performing an additional assessment at the time of transfer; however, this would increase participant burden and radiation exposure without directly contributing to the primary feasibility objectives. The timing of treatment re-initiation, reasons for withdrawal, symptoms and relevant clinical outcomes will be recorded as part of ongoing monitoring.**

 Comment 9

*There are a number of typographical errors which require addressing.*

 We thank the reviewer for highlighting this issue. The manuscript has undergone a further detailed review for spelling, grammar, formatting and typographical errors. All identified errors have been corrected in the revised version.

# Reviewer report: Reviewer response for version 1

**DOI:** 10.3310/nihropenres.15259.r37460

**Published:** 2025-11-10

**Authors:** Daniel Morganstein

**Affiliations:** 1 Chelsea and Westminster NHS Foundation Trust, Imperial College London, London, UK

This protocol describes a feasibility study of stopping growth hormone replacement in adults with GH deficiency.

 It will address questions related to the acceptability of this approach as well as initial safety data to support a future randomised study of stopping vs cessation of growth hormone.

 This is an important question as there is currently limited data to decide if adults with GH data need lifelong treatment or not.

 It would be helpful if the protocol could present any PPIE work that supports the recruitment targets.

Is the study design appropriate for the research question?

Yes

Is the rationale for, and objectives of, the study clearly described?

Yes

Are sufficient details of the methods provided to allow replication by others?

Yes

Are the datasets clearly presented in a useable and accessible format?

Not applicable

Reviewer Expertise:

Endocrine Oncology

I confirm that I have read this submission and believe that I have an appropriate level of expertise to confirm that it is of an acceptable scientific standard.

# Reviewer report: Reviewer response for version 1

**DOI:** 10.3310/nihropenres.15259.r37317

**Published:** 2025-09-30

**Authors:** Giorgio Sodero

**Affiliations:** 1 Universita Cattolica del Sacro Cuore, Rome, Lazio, Italy

The authors have submitted an interesting research protocol concerning the safety and efficacy of GH therapy in adult patients. This is an engaging manuscript; both the study protocol and, above all, the authors’ results deserve to be indexed. However, some clarifications from the authors are required prior to acceptance. Below are my specific comments.

- First, the statement in the abstract *“…no study has consistently demonstrated that GH treatment has a sustained beneficial effect in adults with GHD”* is not entirely accurate. The beneficial effects of GH therapy are well established in both pediatric and adult populations, although I agree that the strongest body of evidence pertains to children. I suggest revising this passage accordingly.
- In the Introduction, I recommend also mentioning GHD diagnosed after childhood; this condition is most often idiopathic.
- The peak value of 9 mU/L is not universally accepted, as significant differences exist across countries. For instance, in my country, the diagnosis of GHD requires two stimulation tests with a peak response below 8, while in others the threshold is below 10, etc. This should be specified.
- The rationale, materials and methods, and study phases are clearly described and, in my opinion, require no further modifications. The ethical committee approval is appropriate.
- In the Discussion, despite persisting uncertainties regarding efficacy in adults, I believe the general efficacy and safety of growth hormone therapy should be emphasized, both in patients with idiopathic GHD and in those with organic forms. The largest prospective study on pediatric patients with endocrinopathies secondary to infectious diseases (10.1097/INF.0000000000004645) demonstrated the incidence of GHD in these conditions and the safety of replacement therapy, although the population included patients younger than 18 years.

Minor improvements in the English language of the manuscript are also needed.

Is the study design appropriate for the research question?

Yes

Is the rationale for, and objectives of, the study clearly described?

Yes

Are sufficient details of the methods provided to allow replication by others?

Yes

Are the datasets clearly presented in a useable and accessible format?

Not applicable

Reviewer Expertise:

Pediatric Endocrinology

I confirm that I have read this submission and believe that I have an appropriate level of expertise to confirm that it is of an acceptable scientific standard, however I have significant reservations, as outlined above.

## References

[1] : Endocrinologic Dysfunctions and Neuropsychiatric Sequelae in Pediatric Patients With a History of Central Nervous System Infection (ENDLESS): A Prospective Monocentric Study. *Pediatric Infectious Disease Journal* .2025;44(4) : 10.1097/INF.0000000000004645310-317 10.1097/INF.0000000000004645 PMC1188882239787566

# Response

**Published:** 2025-09-30

**Authors:** Sherwin Criseno

**Affiliations:** Endocrinology and Metabolism, University Hospitals Birmingham NHS Foundation Trust, Birmingham, England, UK

Dear Dr. Sodero,

 Thank you for your very useful comments and suggestions. We will update the manuscript to incorporate your suggestions. 

 Kind regard,

 Sherwin Criseno
